# Association Between Primary Sclerosing Cholangitis and Faecal Calprotectin Levels in Patients With Inflammatory Bowel Disease: A Systematic Review and Meta-Analysis

**DOI:** 10.7759/cureus.114858

**Published:** 2026-08-20

**Authors:** Nazar Abdelgabar, Eltayeb Osman Elfaki Omer, Alla Mostafa, Eslam Ahmed Abdel Rahman Ahmed, Sumaia Mohammed TalbAllah Alshiekh, Omer Kamal Ahmed Omer, Abdelrahman Elshiekh, Salma Elmansour Abdalla Ismail

**Affiliations:** 1 Gastroenterology and Hepatology, St Mary’s Hospital, Isle of Wight, GBR; 2 Endocrinology, Dr. Sulaiman Al Habib Hospital, Riyadh, SAU; 3 Internal Medicine, King George Hospital, London, GBR; 4 Internal Medicine, Sudan Medical Specialisation Board, Khartoum, SDN; 5 Pathology, Najran University, Najran, SAU; 6 Orthopaedic Surgery, Luton Hospital, Luton, GBR; 7 Gastroenterology, Mersey and West Lancashire Teaching Hospitals, Liverpool, GBR; 8 Gastroenterology, King Fahad Specialist Hospital, Buraydah, SAU

**Keywords:** biomarkers, fecal calprotectin, inflammatory bowel disease, meta-analysis, primary sclerosing cholangitis, ulcerative colitis

## Abstract

Primary sclerosing cholangitis (PSC) is strongly associated with inflammatory bowel disease (IBD), and PSC-associated IBD (PSC-IBD) has a distinct phenotype characterised by extensive, often right-sided colitis, frequent endoscopic and histologic activity despite mild symptoms, and an elevated risk of colorectal neoplasia. Faecal calprotectin (FC) is a validated non-invasive marker of colonic inflammation in IBD, but whether FC concentrations differ between patients with PSC-IBD and IBD without PSC, and how faithfully FC reflects colonic disease activity in PSC-IBD, remain uncertain.

We conducted a systematic review and meta-analysis of observational studies reporting faecal calprotectin concentrations in patients with PSC-IBD and a contemporaneous IBD-without-PSC comparator. Risk of bias was assessed using a structured quality assessment approach. Standardised mean differences (SMD, Hedges' g) in faecal calprotectin were pooled using a DerSimonian-Laird random-effects model with Hartung-Knapp adjustment; medians were converted to means using an established conversion method. Reports arising from a single overlapping cohort were represented once in the quantitative synthesis. Robustness was examined by leave-one-out, mean-difference and ratio-of-means sensitivity analyses, the last of which is better suited to right-skewed data.

Nine reports, arising from four independent cohorts, met the inclusion criteria; three cohorts provided data suitable for meta-analysis. No statistically significant difference in absolute FC between PSC-IBD and IBD without PSC was detected (pooled SMD -0.09, 95% confidence interval -0.41 to 0.22; p=0.56), a finding that was stable across leave-one-out, mean-difference and ratio-of-means sensitivity analyses. Qualitative synthesis indicated that FC may be discordant with colonic disease activity in PSC-IBD: FC was elevated in patients with endoscopically quiescent colitis; its correlation with endoscopic severity relative to IBD without PSC was inconsistent across cohorts; it discriminated mucosal healing with high accuracy, and it correlated closely with biliary calprotectin, while serum (but not faecal) calprotectin was higher in PSC-IBD.

No statistically significant difference in absolute FC concentrations was detected between PSC-IBD and IBD without PSC, although clinically relevant differences cannot be excluded given the wide confidence interval, the small number of cohorts and the very low certainty of the evidence. The available data nonetheless suggest that FC may be elevated out of proportion to colonic inflammation in PSC-IBD and may in part reflect biliary inflammation, a hypothesis that requires confirmation. FC should therefore be interpreted with caution as a marker of colitis activity in PSC-IBD, and adequately powered prospective studies pairing FC with endoscopic, histologic and biliary endpoints are required.

## Introduction and background

Primary sclerosing cholangitis (PSC) is a chronic, progressive cholestatic liver disease characterised by inflammation and fibrosis of the intrahepatic and extrahepatic bile ducts, and it is strongly linked to inflammatory bowel disease (IBD): up to 70-80% of patients with PSC have concomitant IBD, most commonly an ulcerative colitis (UC)-like phenotype [1,2]. The IBD that accompanies PSC (PSC-IBD) is increasingly recognised as a distinct clinical entity rather than simply UC with liver disease. Compared with IBD without PSC, PSC-IBD is more often extensive, exhibits a predilection for the proximal (right) colon with relative rectal sparing and backwash ileitis, and characteristically follows a clinically milder course punctuated by long asymptomatic periods [2,3]. These phenotypic peculiarities complicate the assessment of disease activity using tools validated in conventional IBD.

A central feature of PSC-IBD is that, despite mild symptoms, affected patients frequently harbour ongoing endoscopic and histologic inflammation and carry a several-fold higher risk of colorectal neoplasia than patients with colitis alone [2,4]. Because symptoms systematically underestimate mucosal inflammation in this population, objective, non-invasive markers of disease activity are particularly attractive. Faecal calprotectin (FC), a neutrophil-derived protein measurable in stool, is a well-established surrogate of endoscopically active colonic inflammation in IBD, outperforming C-reactive protein and clinical indices for this purpose [5]. Whether FC behaves in the same way in PSC-IBD is uncertain, however, because the determinants of intestinal inflammation differ from those in conventional IBD, the severity of the underlying liver disease may modulate the colitis [6], and calprotectin has been reported to reach the stool from the inflamed biliary tree as well as from the colon [7].

Individual studies addressing these questions have been small, have used heterogeneous designs and comparators, and have reported FC using different summary statistics, precluding firm conclusions. We therefore conducted a systematic review and meta-analysis to (i) compare FC concentrations between patients with PSC-IBD and IBD without PSC, and (ii) synthesise the available data on the relationship between FC and endoscopic, histologic and biliary disease activity in PSC-IBD.

## Review

Materials and methods

Protocol and Reporting

This systematic review and meta-analysis was reported in accordance with the Preferred Reporting Items for Systematic Reviews and Meta-Analyses (PRISMA) 2020 statement [8]. It was conducted according to a protocol agreed by the review team before study selection and data extraction; the review was not prospectively registered in an international registry such as PROSPERO or the Open Science Framework, which we acknowledge as a limitation. The review question was framed using the Population, Intervention/Exposure, Comparator, Outcome and Study design (PICOS) structure.

Information Sources and Search

PubMed, Embase, Scopus and Web of Science were searched from database inception to the final search date of July 31, 2026. The search combined controlled vocabulary and free-text terms for the concepts of “fecal calprotectin”, “primary sclerosing cholangitis” and “inflammatory bowel disease” (including “ulcerative colitis” and “Crohn's disease”) combined with the Boolean operators AND and OR; the full search strategy for each database is presented in Table 1. Non-English records without an available translation were excluded at full-text review.

**Table 1:** Search strategy for each electronic database. Databases were searched from inception to July 31, 2026. MeSH, Medical Subject Headings; exp, exploded term; ti,ab, title/abstract field; TITLE-ABS-KEY, title, abstract and keyword field; TS, topic field.

Database	Search string	Records retrieved (n)
PubMed	("fecal calprotectin"[tiab] OR "faecal calprotectin"[tiab] OR calprotectin[MeSH]) AND ("primary sclerosing cholangitis"[tiab] OR Cholangitis, Sclerosing[MeSH]) AND ("inflammatory bowel disease"[tiab] OR "ulcerative colitis"[tiab] OR "Crohn disease"[tiab] OR Inflammatory Bowel Diseases[MeSH])	213
Embase	('fecal calprotectin'/exp OR 'calprotectin':ti,ab) AND ('primary sclerosing cholangitis'/exp OR 'sclerosing cholangitis':ti,ab) AND ('inflammatory bowel disease'/exp OR 'ulcerative colitis':ti,ab OR 'Crohn disease':ti,ab)	82
Scopus	TITLE-ABS-KEY("fecal calprotectin" OR "faecal calprotectin") AND TITLE-ABS-KEY("primary sclerosing cholangitis") AND TITLE-ABS-KEY("inflammatory bowel disease" OR "ulcerative colitis" OR "Crohn disease")	47
Web of Science	TS=("fecal calprotectin" OR "faecal calprotectin") AND TS=("primary sclerosing cholangitis") AND TS=("inflammatory bowel disease" OR "ulcerative colitis" OR "Crohn disease")	83
Total		425

Eligibility Criteria

Studies were eligible if they were original, peer-reviewed observational studies (prospective or retrospective cohort, cross-sectional or case-control designs) that reported FC concentrations in patients with PSC-IBD and in a contemporaneous comparator group of patients with IBD without PSC. Both adult and paediatric populations and both UC and Crohn's disease were eligible. Reviews, editorials, guidelines, conference abstracts, letters without primary data, preprints, case reports, and studies lacking an IBD-without-PSC comparator or not reporting FC separately for a PSC-IBD group were excluded. When several reports were derived from the same or an overlapping institutional cohort, they were treated as a single study; the report providing FC as a median with interquartile range (permitting the most reliable reconstruction of the mean and standard deviation) and the largest comparison was used for the quantitative synthesis, and the remaining reports contributed to the qualitative synthesis (Table 2).

**Table 2:** Eligibility criteria according to the PICOS framework. PICOS, Population, Intervention/Exposure, Comparator, Outcome, Study design; IBD, inflammatory bowel disease; IBD-unclassified, inflammatory bowel disease unclassified; PSC, primary sclerosing cholangitis; PSC-IBD, primary sclerosing cholangitis-associated inflammatory bowel disease; UC, ulcerative colitis.

Parameter	Inclusion criteria	Exclusion criteria
Population	Primary sclerosing cholangitis with concomitant IBD (PSC-IBD); adults or children; UC, Crohn's disease or IBD-unclassified	PSC without IBD only; populations in which PSC-IBD cannot be separated
Intervention/exposure	Measurement of faecal calprotectin (any validated assay)	No faecal calprotectin measurement reported
Comparator	Patients with IBD without PSC assessed contemporaneously	No IBD-without-PSC comparator group
Outcomes	Faecal calprotectin concentration and its relationship to endoscopic, histologic, clinical or biliary disease activity	No extractable calprotectin or activity data
Study design	Original observational studies (cohort, case-control, cross-sectional)	Reviews, editorials, guidelines, conference abstracts, letters, preprints, case reports

Study Selection and Data Extraction

Two reviewers independently screened titles and abstracts, assessed full texts against the eligibility criteria, and extracted data, with disagreements resolved by consensus. Of the 19 reports sought for full-text retrieval, seven could not be obtained; these were conference abstracts and meeting proceedings without an accessible full-text article, for which full texts were requested through institutional interlibrary-loan services and, where contact details were available, directly from the corresponding authors, without success. Because these records were abstract-only, they would not have provided the extractable group-level data required for inclusion; nevertheless, the possibility of retrieval bias in a small evidence base is acknowledged. For each included report the following were extracted: first author, year, country, design and setting, population (adult or paediatric; IBD subtype), the number of participants in each group, FC assay, the metric and summary statistic reported for FC (mean with standard deviation (SD), or median with interquartile range (IQR) or range), and all reported data relating FC to endoscopic, histologic, clinical or biliary disease activity.

Risk of Bias

The methodological quality of each included report was appraised independently by two reviewers using the Newcastle-Ottawa Scale (NOS), with disagreements resolved by consensus. The scale was adapted to the relevant observational design so that, for cross-sectional and case-control studies, the Selection domain assessed the representativeness and definition of the PSC-IBD and comparator groups and the ascertainment of PSC and IBD diagnoses, the Comparability domain assessed whether the groups were balanced for or the analysis adjusted for colitis activity, extent and treatment, and the Outcome domain assessed the objectivity of FC measurement and the completeness of data [9]. Reports awarded seven or more of a maximum of nine stars were considered of good quality, five to six fair, and fewer than five poor. The certainty of the pooled evidence for the primary outcome was rated using the Grading of Recommendations Assessment, Development and Evaluation (GRADE) approach, considering risk of bias, inconsistency, indirectness, imprecision and publication bias [10].

Statistical Analysis

The primary outcome was the difference in FC concentration between PSC-IBD and IBD-without-PSC groups. Because FC assays, units and baseline concentrations varied across studies, effect sizes were expressed as standardised mean differences (SMD; Hedges' g), with a positive value indicating higher FC in PSC-IBD. For studies reporting FC as a median with IQR, the mean and SD were estimated using the distribution-based methods of Wan et al. [11]. SMDs were pooled using a DerSimonian-Laird random-effects model [12]; because the number of studies was small, confidence intervals and the test of the overall effect were derived using the Hartung-Knapp adjustment, and the more conservative of the resulting intervals was reported [13]. Between-study heterogeneity was quantified using the I-squared statistic and the between-study variance (tau-squared); because heterogeneity cannot be estimated reliably from only three cohorts, I-squared was interpreted with caution and not taken as evidence of true homogeneity. Robustness was examined by leave-one-out analysis and by expressing the effect as a raw mean difference in micrograms per gram. Because FC is markedly right-skewed and two of the three pooled cohorts reported medians with IQRs that required conversion to means, a further sensitivity analysis used the ratio-of-means method, which operates on the natural-log scale and does not assume normality. Given the small number of cohorts, the subgroup analysis by comparator (ulcerative colitis only versus mixed IBD) was regarded as exploratory. A two-sided p-value below 0.05 was considered statistically significant. Analyses were performed in Python (SciPy and NumPy) [14]. Reports that did not provide extractable group-level FC summary statistics were synthesised narratively.

Results

**Figure 1:**
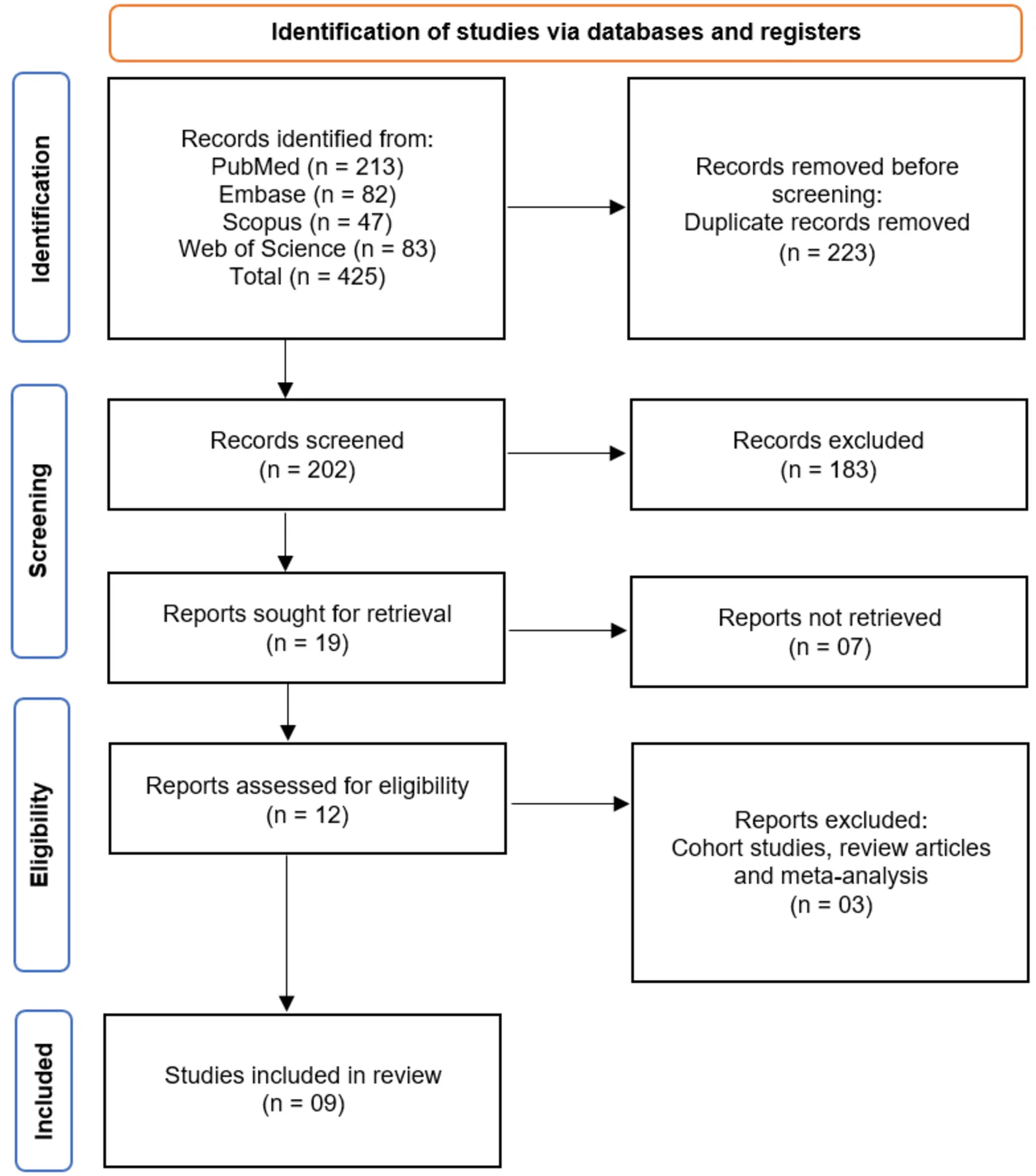
PRISMA 2020 flow diagram of study identification, screening and inclusion. PRISMA, Preferred Reporting Items for Systematic Reviews and Meta-Analyses.

The study selection process is summarised in the Preferred Reporting Items for Systematic Reviews and Meta-Analyses (PRISMA) flow diagram (Figure 1). A total of 425 records were identified through database searching (PubMed, n=213; Embase, n=82; Scopus, n=47; Web of Science, n=83). After 223 duplicate records were removed, 202 records were screened by title and abstract, of which 183 were excluded. Nineteen reports were sought for retrieval; seven could not be retrieved (Materials and Methods), leaving 12 reports assessed in full text for eligibility. Three reports were excluded at this stage (cohort studies without an eligible comparator, review articles and meta-analyses), and nine reports, arising from four independent cohorts, were included in the systematic review [7,15-22]. Three cohorts provided group-level FC summary statistics suitable for meta-analysis [7,16,17]. One cohort (paediatric) reported FC only stratified by mucosal-healing status rather than as an overall group summary and could not be pooled [15]. A further six reports originated from a single centre and drew on an overlapping patient population [17-22]; to avoid double-counting participants, these were treated as one cohort, and the report providing FC as a median with interquartile range in the largest sample was used for the quantitative synthesis [17], with the remaining reports contributing to the qualitative synthesis. Throughout, we distinguish between the nine included reports and the four independent cohorts from which they arise.

The characteristics of the included reports are presented in Table 3, and their FC data and principal findings are presented in Table 4. The included cohorts comprised patients from the United Kingdom [7,16], Canada [15] and a single German centre [17-22]. Designs included a prospective surveillance cohort [7], a prospective paediatric cohort [15], a case-control study [16] and a series of cross-sectional biomarker reports [17-22]; all but the paediatric cohort enrolled adults. FC was measured by enzyme-linked immunosorbent assay in all studies that specified the method, most commonly the Buhlmann assay. Among the three cohorts contributing to the meta-analysis, FC was reported as a median with IQR in two [7,17] and as a mean with SD in one [16]. Baseline colitis activity, disease extent and concurrent treatment differed across, and were incompletely reported within, the included cohorts; where reported, comparisons were frequently made within endoscopically quiescent disease (for example, the surveillance cohort contrasted PSC-IBD and UC patients with an endoscopic index of 0-1 [7]) and the single-centre cohort comprised predominantly patients with low FC consistent with quiescent colitis. This variation in disease activity and treatment is a potential confounder of any between-group difference in FC.

**Table 3:** Characteristics of the included reports. Reports sharing a cohort letter draw on the same institutional population; cohort D (six reports) contributed to the meta-analysis through Matysik et al., 2025, only [17]. CD, Crohn's disease; IBD, inflammatory bowel disease; IBD-U, inflammatory bowel disease unclassified; n, number of patients; PSC, primary sclerosing cholangitis; PSC-IBD, primary sclerosing cholangitis-associated inflammatory bowel disease; UC, ulcerative colitis; UK, United Kingdom.

Study	Cohort	Country	Design	Population	PSC-IBD, n	Comparator (n)
Pavlidis et al., 2022 [7]	A	UK	Prospective cohort	Adults	20	UC (20)
Bjarnason et al., 2015 [16]	B	UK	Case-control	Adults	16	UC (55); CD (55)
Ricciuto et al., 2018 [15]	C	Canada	Prospective cohort	Children	37	UC/IBD-U (50)
Matysik et al., 2025 [17]	D	Germany	Cross-sectional	Adults	21	IBD (80)
Bajraktari et al., 2024 [18]	D	Germany	Cross-sectional	Adults	11	IBD (55)
Elger et al., 2025 [19]	D	Germany	Cross-sectional	Adults	13	IBD (57)
Elger et al., 2026 [20]	D	Germany	Cross-sectional	Adults	35	IBD (74)
Elger et al., 2026 [21]	D	Germany	Cross-sectional	Adults	33	IBD (77)
Elger et al., 2026 [22]	D	Germany	Cross-sectional	Adults	13	IBD (57)

**Table 4:** Faecal calprotectin data and principal findings of the included reports. AUC, area under the receiver operating characteristic curve; CD, Crohn's disease; FC, faecal calprotectin; IBD, inflammatory bowel disease; IQR, interquartile range; MH, mucosal healing; PSC, primary sclerosing cholangitis; PSC-IBD, primary sclerosing cholangitis-associated inflammatory bowel disease; SD, standard deviation; UC, ulcerative colitis.

Study	PSC-IBD FC	Comparator FC	Principal finding
Pavlidis et al., 2022 [7]	323 (95-788)	307 (57-842) (UC)	FC raised despite quiescent colitis; correlates with biliary calprotectin; predicts biliary complications
Bjarnason et al., 2015 [16]	428 ± 351	501 ± 656 (UC)	FC comparable to UC and CD despite histologically milder, right-sided colitis
Ricciuto et al., 2018 [15]	By MH status	by MH status	FC discriminates mucosal healing (AUC 0.94); symptoms do not; subclinical activity in clinical remission
Matysik et al., 2025 [17]	38 (17-146)	57 (25-158) (IBD)	FC similar between groups; not correlated with serum/faecal sterols
Bajraktari et al., 2024 [18]	by PSC group	49 (IBD)	FC not correlated with serum galectin-3
Elger et al., 2025 [19]	34 (0-222)	58 (0-1616) (IBD)	FC not correlated with the long-to-very-long-chain ceramide ratio
Elger et al., 2026 [20]	41 (0-999)	55 (0-3402) (IBD)	Faecal FC similar, but serum calprotectin higher in PSC-IBD (AUC 0.70)
Elger et al., 2026 [21]	48 (0-999)	54 (0-3889) (IBD)	FC not correlated with serum soluble CD137
Elger 2026 [22]	By PSC group	211 ± 388 (IBD)	FC not correlated with serum phosphatidylcholine species

Risk-of-bias assessments for all nine included reports are summarised in Table 5. Overall methodological quality ranged from fair to good. The prospective paediatric cohort scored highest, reflecting its prospective design, blinded outcome assessment and relatively large sample for a rare condition [15], while the prospective surveillance-based cohort was also judged to be of good quality [7]. The case-control study and the cross-sectional biomarker reports were rated fair, principally reflecting small PSC-IBD sample sizes, single-centre recruitment, and limited comparability between groups that differed in colitis extent, activity and treatment [16-22]. No report was rated poor.

**Table 5:** Risk-of-bias assessment of the included reports (Newcastle-Ottawa Scale). The Selection domain assessed group definition and diagnostic ascertainment; the Comparability domain assessed balance of, or adjustment for, colitis activity, extent and treatment; the Outcome domain assessed the objectivity of faecal calprotectin measurement and completeness of data [9]. Reports scoring ≥7 stars were rated good, 5-6 fair, and <5 poor.

Study	Selection (/4)	Comparability (/2)	Outcome (/3)	Total (/9)	Quality
Pavlidis et al., 2022 [7]	3	1	3	7	Good
Bjarnason et al., 2015 [16]	3	1	2	6	Fair
Ricciuto et al., 2018 [15]	4	2	3	9	Good
Matysik et al., 2025 [17]	2	1	2	5	Fair
Bajraktari et al., 2024 [18]	2	1	2	5	Fair
Elger et al., 2025 [19]	2	1	2	5	Fair
Elger et al., 2026 [20]	2	1	2	5	Fair
Elger et al., 2026 [21]	2	1	2	5	Fair
Elger et al., 2026 [22]	2	1	2	5	Fair

**Figure 2:**
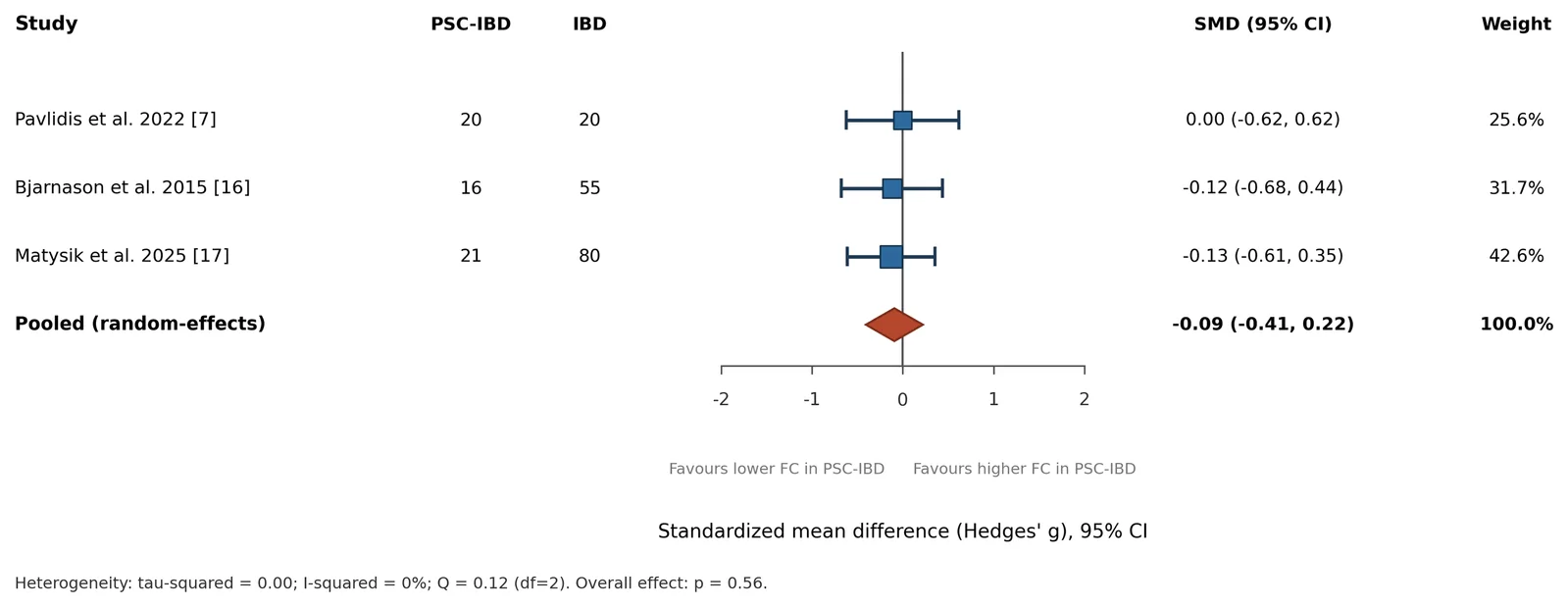
Forest plot of the standardised mean difference in faecal calprotectin between PSC-IBD and IBD without PSC (random-effects model). The pooled estimate is not statistically significant. IBD, inflammatory bowel disease; PSC, primary sclerosing cholangitis; PSC-IBD, primary sclerosing cholangitis-associated inflammatory bowel disease.

Three independent cohorts contributing 57 patients with PSC-IBD and 155 with IBD without PSC were pooled for the comparison of absolute FC concentrations (Figure 2). The individual standardised mean differences (SMD) were 0.00 [7], -0.12 [16] and -0.13 [17]. The pooled random-effects estimate showed no statistically significant difference in FC between PSC-IBD and IBD without PSC (SMD -0.09, 95% confidence interval (CI) -0.41 to 0.22; p=0.56). Although the I-squared statistic was 0% (tau-squared=0.00, Cochran Q=0.12, df=2), this should not be interpreted as evidence of true homogeneity, because between-study heterogeneity cannot be estimated reliably from only three cohorts.

**Figure 3:**
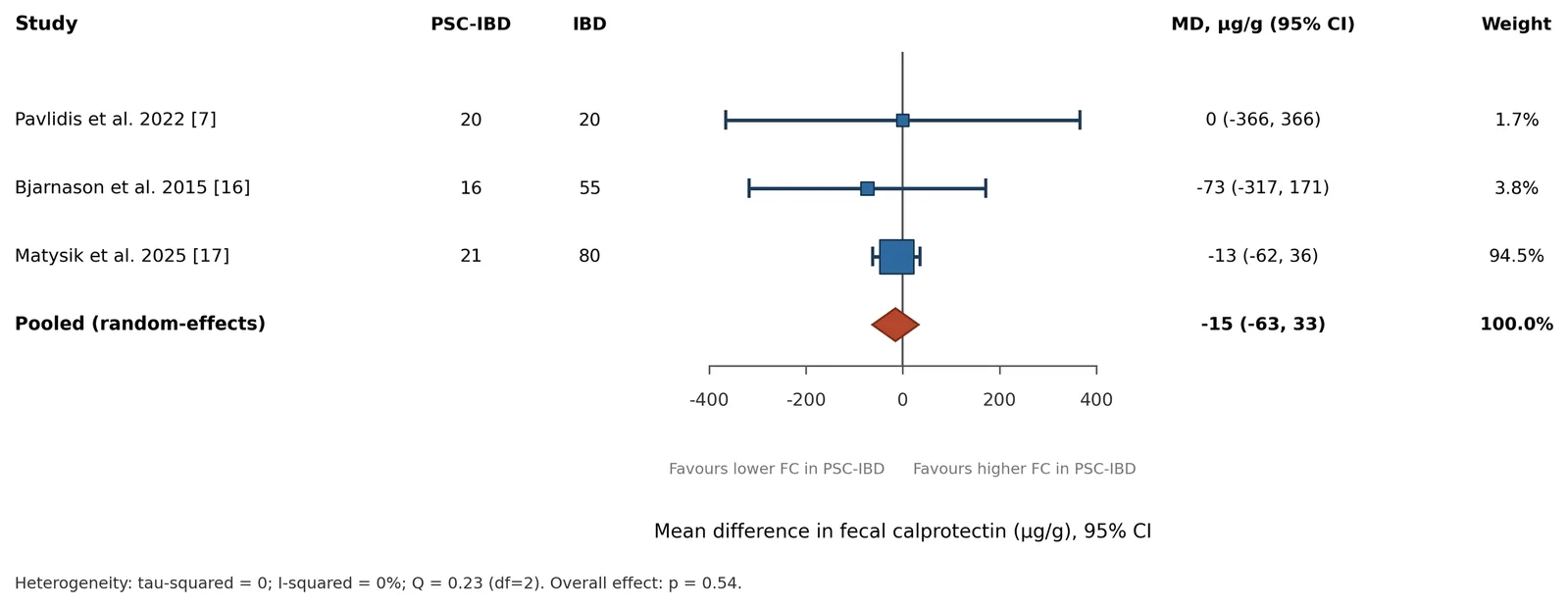
Sensitivity analysis expressing the effect as a raw mean difference in faecal calprotectin (µg/g), dominated by the cohort with the smallest variance. IBD, inflammatory bowel disease; PSC-IBD, primary sclerosing cholangitis-associated inflammatory bowel disease; MD, mean difference.

The absence of a detectable difference was consistent across several sensitivity analyses. Expressed as a raw mean difference in native units, FC was 15.1 microg/g lower in PSC-IBD than in IBD without PSC, without statistical significance (95% CI -62.8 to 32.6; p=0.54; I-squared=0%; Figure 3); this analysis was dominated by the single cohort with the smallest variance, illustrating why the standardised mean difference was pre-specified as the primary measure.

**Figure 4:**
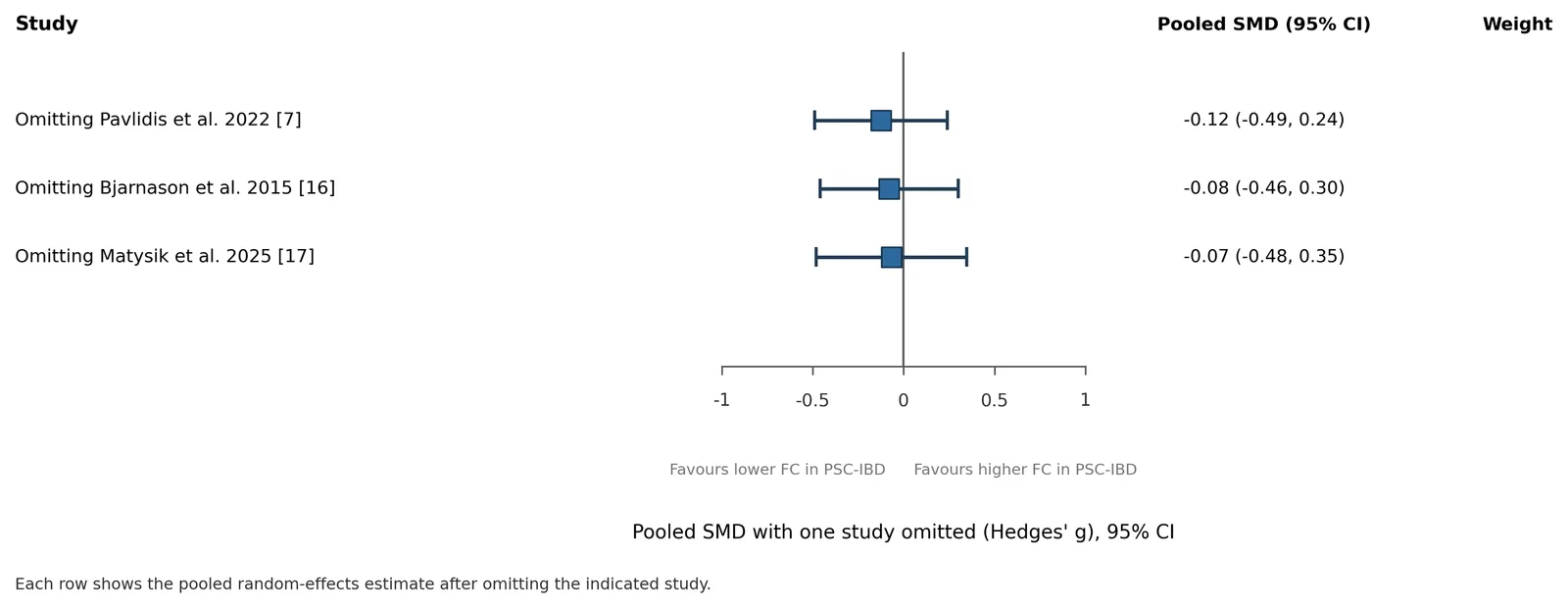
Leave-one-out sensitivity analysis. Each row shows the pooled standardised mean difference after omitting the indicated study; all estimates remain non-significant. SMD, standardised mean difference.

Because FC is markedly right-skewed, a ratio-of-means analysis on the natural-log scale, which does not assume normality, was also performed and yielded a pooled ratio of 0.87 (95% CI 0.59 to 1.28; p=0.49; I-squared=0%), consistent in direction and in its non-significance with the primary analysis. In leave-one-out analysis, the pooled SMD ranged only from -0.07 to -0.12 and remained non-significant regardless of which cohort was omitted (Figure 4). In an exploratory subgroup analysis by comparator, in which one subgroup comprised two cohorts and the other effectively a single cohort and which was therefore markedly underpowered, the pooled SMD was -0.07 (95% CI -0.48 to 0.35) with an ulcerative colitis-only comparator and -0.13 (95% CI -0.61 to 0.35) with a mixed-IBD comparator; the non-significant subgroup difference (p=0.85) should be regarded as exploratory rather than as evidence of absence of a difference.

The certainty of the pooled evidence for the difference in FC between PSC-IBD and IBD without PSC was rated as very low according to the GRADE approach (Table 6), reflecting the observational design of the included studies, their overall risk of bias, and imprecision arising from the small number of cohorts and a wide confidence interval that crossed the null.

**Table 6:** GRADE certainty-of-evidence assessment for the primary outcome ¹Downgraded for observational design and predominantly fair methodological quality. ²Downgraded for the small number of cohorts and a wide confidence interval crossing the null. GRADE certainty: observational studies start at low and were downgraded to very low. GRADE, Grading of Recommendations Assessment, Development and Evaluation; IBD, inflammatory bowel disease; PSC, primary sclerosing cholangitis; PSC-IBD, primary sclerosing cholangitis-associated inflammatory bowel disease.

Outcome	Studies (participants)	Risk of bias	Inconsistency	Indirectness	Imprecision	Certainty
Faecal calprotectin, PSC-IBD vs. IBD without PSC	3 cohorts (212)	Serious¹	Not serious	Not serious	Serious²	⊕⊖⊖⊖ Very low

Faecal Calprotectin and Colonic Disease Activity

Although absolute FC concentrations did not distinguish PSC-IBD from IBD without PSC, the included studies suggested that FC may be discordant with the degree of colonic inflammation in PSC-IBD. In the prospective surveillance cohort, patients with PSC-IBD and endoscopically quiescent colitis (ulcerative colitis endoscopic index of severity (UCEIS) 0-1) had markedly higher FC than patients with UC and comparably quiescent disease (median 279 vs 30 microg/g; p=0.015), and the correlation between FC and endoscopic severity was weaker in PSC-IBD (r=0.596) than in UC (r=0.821) [7]. In the same cohort FC correlated strongly with biliary calprotectin measured in paired samples (r=0.898), and an elevated FC (>250 microg/g) predicted subsequent biliary complications (hazard ratio 16.39, 95% CI 2.98-90.25) rather than colitis flares, raising the possibility of a biliary contribution to the faecal signal; this evidence, however, derives from a single cohort and is hypothesis-generating [7]. Consistent with disproportionate inflammatory output, the case-control study found FC concentrations in PSC-IBD (428 plus/minus 351 microg/g) that were statistically indistinguishable from those in UC (501 plus/minus 656 microg/g) and Crohn's disease (476 plus/minus 571 microg/g), despite the colitis of PSC being histologically milder and predominantly right-sided [16].

Evidence on the FC-endoscopy relationship was, however, mixed and cohort-specific. In contrast to the weaker correlation seen in the adult surveillance cohort [7], the paediatric cohort found that FC correlated with endoscopic activity to a numerically similar or stronger degree in PSC-IBD than in controls (r=0.78 vs 0.68; p=0.40) and discriminated mucosal healing with excellent accuracy in PSC-IBD (area under the curve 0.94; optimal cut-off 93 microg/g; sensitivity 100%, specificity 92%), markedly outperforming symptom-based scoring [15]. Nonetheless, children with PSC-IBD judged to be in clinical remission had substantially greater odds of active endoscopic disease than controls in clinical remission (odds ratio 5.9, 95% CI 1.6-21.5), and within the subgroup already in biochemical remission (FC <100 microg/g) endoscopic activity no longer differed between groups [15]. In the single-centre cohort, FC was similar between PSC-IBD and IBD across successive biomarker reports (medians 34-48 vs 54-58 microg/g) [17-22] and did not correlate with the candidate serum or lipid markers examined, including galectin-3, ceramides, phosphatidylcholine species and adiponectin [18-22]. Notably, in the same cohort, serum (as opposed to faecal) calprotectin was higher in PSC-IBD than in IBD (area under the curve 0.70), paralleling a systemic and possibly biliary inflammatory signal rather than colonic activity [20].

Discussion

This systematic review and meta-analysis examined whether faecal calprotectin concentrations differ between patients with PSC-associated IBD and those with IBD without PSC, and how FC relates to colonic disease activity in PSC-IBD. Pooling three independent cohorts, we detected no statistically significant difference in absolute FC between the two groups (SMD -0.09, 95% CI -0.41 to 0.22), a result that was stable across leave-one-out, mean-difference and ratio-of-means sensitivity analyses. This absence of a detectable difference should not, however, be read as evidence of equivalence: the confidence interval remained wide, only three cohorts contributed, and the certainty of the evidence was very low, so clinically relevant differences cannot be excluded. Alongside this quantitative finding, the qualitative synthesis suggested a clinically relevant pattern, namely that FC in PSC-IBD may be elevated out of proportion to endoscopically quiescent colitis and may, in at least one cohort, partly reflect biliary rather than colonic inflammation [7,15,16].

The observation that patients with PSC-IBD and endoscopically quiescent colitis nonetheless exhibited substantially higher FC than comparably quiescent patients with UC is difficult to reconcile with a purely colonic origin of the signal [7]. Two explanations are compatible with the data. First, the colitis of PSC-IBD is characteristically right-sided and, although histologically often mild, may generate a disproportionate neutrophilic flux into the lumen, consistent with the finding that FC in PSC-IBD equalled that of conventional UC and Crohn's disease despite milder histology [16]. Second, calprotectin may enter the stool from the inflamed biliary tree: in one cohort, paired faecal and biliary calprotectin were tightly correlated and an elevated FC presaged biliary complications rather than colitis flares [7]. Because this direct evidence for a biliary source comes predominantly from a single cohort, it should be regarded as a hypothesis to be tested rather than an established mechanism; if confirmed, it would provide a plausible explanation, through the gut-liver axis, for an elevated FC when the colon is endoscopically and histologically quiet.

Interpretation of FC as a marker of colitis activity in PSC-IBD is complicated by the fact that the two studies reporting a direct FC-endoscopy comparison disagreed. Whereas the adult surveillance cohort found a weaker correlation between FC and endoscopic severity in PSC-IBD than in UC [7], the paediatric cohort found a numerically stronger correlation in PSC-IBD, with no significant between-group difference, and showed that FC discriminated mucosal healing with high accuracy while symptom-based indices did not [15]. The evidence that FC is a poorer marker of colonic inflammation in PSC-IBD is therefore not consistent. What can be stated more cautiously is that a low FC remains reassuring and is associated with mucosal healing, whereas an elevated FC is less specific in PSC-IBD and may reflect subclinical proximal colonic inflammation, biliary inflammation, or both. Given that PSC-IBD carries a several-fold increased risk of colorectal neoplasia driven by cumulative, often subclinical, inflammation, and that symptoms are inadequate for gauging mucosal status, an unexpectedly elevated FC in an otherwise well PSC-IBD patient may reasonably prompt objective evaluation of the colon and consideration of biliary disease; this suggestion, however, rests on limited, very-low-certainty evidence and requires prospective confirmation before it can inform routine practice.

Our results sit within a broader literature on the distinctive biology of PSC-IBD. Endoscopic and histologic studies have repeatedly shown that patients with UC and PSC in apparent clinical remission harbour more subclinical inflammation than patients with UC alone, particularly in the proximal colon, providing a mechanistic explanation for their right-sided predominance of neoplasia [4]. The recognition of PSC-IBD as a phenotypically distinct form of IBD, more extensive, more often rectal-sparing, with backwash ileitis and a milder symptomatic course, is well established across cohorts and reviews [2,3], and the severity of the underlying PSC has itself been linked to the behaviour of the associated colitis [6]. Against this background, FC remains a useful non-invasive marker in PSC-IBD, but its interpretation must be tailored to the phenotype rather than transposed uncritically from conventional IBD, where FC is a robust surrogate of endoscopic activity [5].

The single-centre biomarker reports that constitute the largest block of included evidence are internally consistent: across successive analyses of galectin-3, ceramides, phosphatidylcholine species, sterols and adiponectin, faecal calprotectin was similar between PSC-IBD and IBD and was not correlated with any of these candidate markers, whereas serum calprotectin in the same cohort was higher in PSC-IBD than in IBD [17-22,20]. A divergence between an unchanged faecal signal and an elevated systemic signal would be compatible with the hypothesis that the additional calprotectin in PSC-IBD arises from biliary and systemic inflammation rather than from the colonic mucosa, but, as noted, this remains to be demonstrated directly.

Several questions follow. If FC in PSC-IBD is partly biliary in origin, it might serve not only as a marker of colitis but as a candidate marker of biliary inflammation and PSC activity, a possibility supported by the association between elevated FC and subsequent biliary complications in one cohort [7] and one that would be valuable given the paucity of non-invasive markers of PSC progression. Prospective studies that pair serial FC measurement with contemporaneous endoscopic scoring, segmental histology, cholangiographic assessment and clinical outcomes are needed to partition the faecal signal, to define thresholds specific for colonic versus biliary inflammation, and to determine whether FC trajectories carry prognostic information. Until such evidence is available, FC in PSC-IBD is best regarded as a sensitive but non-specific integrator of gut and, possibly, biliary inflammation whose elevation warrants interpretation in the full clinical, endoscopic and cholestatic context.

Limitations

This review has several limitations, most of which reflect the state of the underlying evidence. First, and most importantly, only three independent cohorts (57 patients with PSC-IBD and 155 with IBD without PSC) contributed to the meta-analysis, so the pooled estimate, the subgroup analysis and the estimate of heterogeneity are all inherently unstable; the I-squared of 0% should not be taken as evidence that the cohorts are truly homogeneous, and the absence of a statistically significant difference must not be equated with equivalence. Second, FC is markedly right-skewed and two of the three pooled cohorts reported medians with IQRs that required conversion to means under an assumption of approximate normality; although we mitigated this by preferring reports with interquartile ranges and by adding a ratio-of-means sensitivity analysis better suited to skewed data, residual bias from the conversion cannot be excluded. Third, six of the nine included reports originated from a single centre with an overlapping patient population, which we treated as one cohort to avoid double-counting but whose influence on the qualitative synthesis cannot be excluded, and two of the remaining cohorts shared an institution. Fourth, seven of 19 reports sought could not be retrieved; although these appeared to be abstract-only records lacking extractable data, the possibility of retrieval bias cannot be fully excluded. Fifth, the compared groups differed in, and often incompletely reported, baseline colitis activity, disease extent and treatment, all of which strongly influence FC and may therefore confound between-group comparisons. Sixth, because primary studies seldom reported FC jointly with paired endoscopic, histologic and biliary endpoints, the evidence for discordance and for a biliary contribution rests on a limited number of observations and remains hypothesis-generating. Consistent with these constraints, the GRADE certainty of the pooled estimate was very low. These limitations temper our conclusions and underscore the need for larger, standardised, prospective studies.

## Conclusions

In patients with PSC-associated IBD, no statistically significant difference in absolute faecal calprotectin was detected between PSC-IBD and IBD without PSC, although clinically relevant differences cannot be excluded given the wide confidence interval, the small number of contributing cohorts and the very low certainty of the evidence. The available data nonetheless suggest that FC may be discordant with the degree of colonic inflammation in PSC-IBD, being elevated out of proportion to often quiescent colitis and possibly reflecting, in part, biliary inflammation, a hypothesis underscored by the finding of elevated serum but not faecal calprotectin in this population and requiring prospective confirmation. A low FC is likely to remain a reassuring marker of mucosal healing in PSC-IBD, but an elevated FC should not be interpreted as evidence of active colitis without corroborating endoscopic, histologic or biliary assessment. Adequately powered prospective studies that pair serial FC with endoscopic, histologic and biliary endpoints are needed to define how this accessible biomarker can best be used in PSC-IBD.
